# A Novel Brain-Targeting Nanoparticle Loaded with Biatractylolide and Its Protective Effect on Alzheimer’s Disease

**DOI:** 10.3390/ph18060809

**Published:** 2025-05-28

**Authors:** Qianmei Hu, Candi Liu, Jiawang Tan, Jixiang Wang, Hao Yang, Yi Liu, Haochu Mao, Zixuan Jiang, Xing Feng, Xiaojun Tao

**Affiliations:** 1 Key Laboratory of Model Animals and Stem Cell Biology in Hunan Province, Hunan Normal University Health Science Center, Changsha 410013, China; 2 Key Laboratory of Study and Discovery of Small Targeted Molecules of Hunan Province, Department of Pharmacy, Hunan Normal University Health Science Center, Changsha 410013, China; 3 Engineering Research Center of Reproduction and Translational Medicine of Hunan Province, Hunan Normal University Health Science Center, Changsha 410013, China; 4 Institute of Interdisciplinary Studies, Hunan Normal University, Changsha 410081, China; 5 Hunan Drug Inspection Center, Changsha 410011, China

**Keywords:** biatractylolide, Alzheimer’s disease, pullulan polysaccharide, nanoparticle, brain targeting

## Abstract

**Background:** To enhance the bioavailability and neuroprotective efficacy of biatractylolide against Alzheimer’s disease by developing a novel Tween-80-modified pullulan–chenodeoxycholic acid nanoparticle as a delivery vehicle. **Methods**: Chenodeoxycholic acid (CDCA) was chemically conjugated to pullulan to yield hydrophobically modified pullulan (PUC), onto which polysorbate 80 (Tween-80) was subsequently adsorbed. The PUC polymers with CDCA substitution levels were analyzed by ^1^H NMR spectroscopy. Nanoparticles were fabricated via the dialysis method and characterized by transmission electron microscopy and dynamic light scattering for morphology, size, and surface charge. In vitro neuroprotection was assessed by exposing SH-SY5Y and PC12 cells to 20 µM Aβ_25-35_ to induce cytotoxicity, followed by pretreatment with biatractylolide-loaded PUC (BD-PUC) nanoparticle solutions at various biatractylolide concentrations. The in vivo brain-targeting capability of both empty PUC and BD-PUC particles was evaluated using a live imaging system. **Results**: The ^1^H NMR analysis confirmed three distinct CDCA substitution degrees (8.97%, 10.66%, 13.92%). Transmission electron microscopy revealed uniformly dispersed, spherical nanoparticles. Dynamic light scattering measurements showed a hydrodynamic diameter of ~200 nm and a negative zeta potential. Exposure to 20 µM Aβ_25-35_ significantly reduced SH-SY5Y and PC12 cell viability; pretreatment with BD-PUC nanoparticles markedly enhanced cell survival rates and preserved cellular morphology compared to cells treated with free biatractylolide. Notably, the cytoprotective effect of BD-PUC exceeded that of the free drug. In vivo imaging demonstrated that both empty PUC and Tween-80-adsorbed BD-PUC nanoparticles effectively accumulated in the brain. **Conclusions**: The protective effect of BD-PUC on SH-SY5Y and PC12 cells induced by Aβ_25-35_ was higher than free biatractylolide solution, and the BD-PUC nanosolution modified with Tween-80 showed a brain-targeting effect.

## 1. Introduction

Alzheimer’s disease (AD) is a neurodegenerative disorder predominantly affecting the elderly population [1]. The hallmark features of AD include a significant reduction in choline acetyltransferase (ChAT) and acetylcholine (Ach) levels as well as the accumulation of senile plaques in the brain, which are composed of amyloid-beta (Aβ) proteins. Over the past decades, a great deal of research has been conducted to develop and refine treatments for Alzheimer’s disease. However, the development of new clinical therapies for AD has been slow and has often failed to achieve significant success [2,3].

AD exhibits substantial heterogeneity in both pathophysiology and therapeutic response, challenging the premise of universal amyloid-targeting therapies [4]. Clinical trials revealed divergent outcomes: amyloid clearance may yield limited cognitive benefits in select patients yet prove ineffective or detrimental in others, reflecting the disease’s biological complexity [5]. This variability aligns with the frequent failure of amyloid-centric therapies to achieve consistent clinical improvements, even when amyloid plaque reduction is achieved. Notably, most trials demonstrated minimal impact on disease progression or sustained cognitive enhancement, as evidenced by unchanged daily functioning and quality-of-life metrics despite amyloid reduction [6]. These observations imply that amyloid-focused strategies inadequately address parallel pathological processes—tau aggregation, synaptic dysfunction, neuroinflammation—which may independently or synergistically drive neurodegeneration. Safety concerns further limit amyloid-targeting approaches, with brain edema, microhemorrhages, and amyloid-related imaging abnormalities disproportionately affecting older populations with cerebrovascular comorbidities, often necessitating trial discontinuation or protocol modifications. These limitations have prompted a strategic shift toward multimodal interventions targeting AD’s multifactorial etiology. Current research prioritizes the combined modulation of tau pathology, neuroinflammatory pathways, mitochondrial dysfunction, and vascular contributions, reflecting recognition that effective disease modification requires the simultaneous engagement of interconnected pathological networks rather than isolated amyloid suppression [7].

Several plants were identified as containing active components that inhibit acetylcholinesterase [8,9]. This indicates that herbal medicine is one of the most valuable resources for acetylcholinesterase inhibitors [10]. In our research group, we successfully isolated a novel diterpenoid, biatractylolide (BD), from the Chinese herb *Atractylodes macrocephala*. This compound was shown to be a potent cholinesterase inhibitor with therapeutic potential for AD, as demonstrated by molecular docking studies and both in vivo and in vitro experiments [11,12]. However, BD is lipophilic and exhibits low bioavailability.

To effectively treat AD, the blood–brain barrier (BBB) must be addressed. The blood–brain barrier is composed of brain capillary endothelial cells, astrocytes, and pericytes. The tightly arranged connections between the cells form a continuous single-layer cellular barrier that controls and prevents the free flow of substances through the blood and brain tissue [13,14]. In order to deliver drugs through the BBB for the treatment of AD, the use of nanodelivery systems is considered. Nanoparticles can enhance the solubility of insoluble drugs due to their large specific surface area [15,16,17]. By modifying the surface of nanoparticles with different biomaterials and chemicals, drugs can be localized and delivered to the brain, overcoming the barrier to BBB penetration [18].

Pullulan is a natural macromolecular, straight-chain polysaccharide that is soluble in water, safe, and non-toxic, with advantageous plasticity and film-forming properties [19]. There are many hydroxyl groups on its molecular chain, and the groups of some hydrophobic substances can be attached to the polysaccharide backbone by esterification or carboxymethylation reactions with these hydroxyl groups to form amphiphilic polymers; it finds wide-ranging applications in the pharmaceutical, food, cosmetic, and agrochemical industries [20,21]. CDCA is a hydrophobic compound derived from animal gallbladders and is characterized by high biocompatibility and low toxicity [22]. The carboxyl group in this structure can combine with the hydroxyl group in pullulan to form an amphiphilic polymer.

The Tween-80-modified nanoparticles can be adsorbed to ApoE in plasma [23,24] and are recognized and taken up by endothelial cells after interacting with the low-density lipoprotein (LDL) receptor of the blood–brain barrier endothelial cells and diffuse into the brain. Nanoparticles modified with Tween-80 appear to have brain-targeting properties [25,26,27].

In this study, a pullulan polymer modified with CDCA was selected as the carrier. The compound biatractylolide was physically embedded to form polymeric nanoparticles. And Tween-80 was adsorbed onto the nanoparticles to form brain-targeting nanoparticles.

The present study proposed a novel therapeutic strategy with some distinct advantages over conventional amyloid-targeting immunotherapies. It employed a unique mechanism of action through biatractylolide, a natural herbal compound that exerts neuroprotective effects via dual pathways: the regulation of PI3K-Akt-GSK3β-dependent pathways and acetylcholinesterase inhibition [11,12] rather than focusing solely on Aβ reduction. This pharmacological profile suggests potential therapeutic efficacy even in cases without significant amyloid load reduction, differentiating it from traditional Aβ-centric approaches. The implementation of a nanoparticle-based delivery system specifically addresses historical challenges in cerebral drug administration by enhancing biatractylolide’s bioavailability and facilitating BBB penetration, thereby overcoming the limited brain exposure that compromised previous therapeutic attempts. The targeted delivery mechanism may reduce systemic drug distribution, potentially mitigating the off-target effects that have hindered clinical translation of conventional amyloid-targeting therapies. These combined features—multimodal neuroprotection, optimized cerebral biodistribution, and localized pharmacological action—collectively aim to expand the therapeutic window while improving both safety profiles and clinical outcomes.

## 2. Results

### 2.1. Characteristics of Nanoparticles

#### 2.1.1. IR Spectroscopy of PUC Polymers

**Figure 1:**
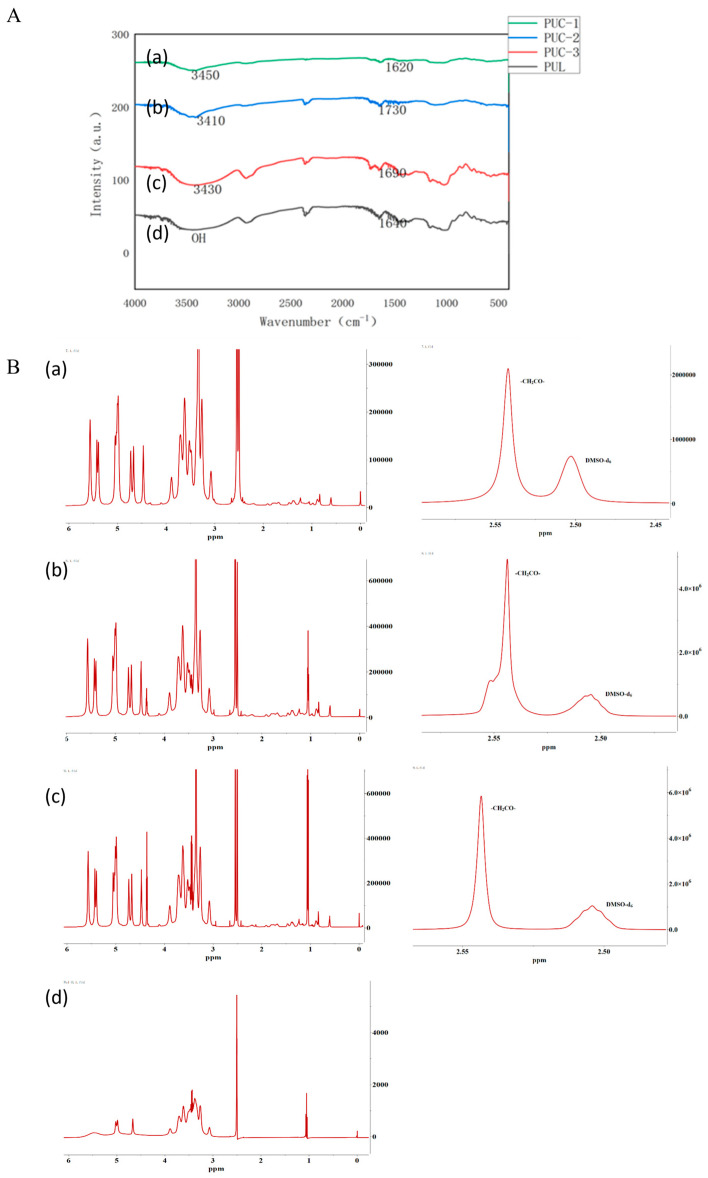
(**A**). PUC polymer synthesis using infrared spectrophotometry, (**a**) pullulan, (**b**) 0.13 PUC polymer, (**c**) 0.15 PUC polymer, (**d**) 0.18 PUC polymer. (**B**). NMR detection of PUC polymer synthesis, (**a**) 0.13 PUC polymer, (**b**) 0.15 PUC polymer, (**c**) 0.18 PUC polymer, (**d**) pullulan.

The IR spectra of the PUC polymers with varying degrees of substitution (Figure 1A) revealed an absorption peak around 3400 cm^−1^, characteristic of the unreacted hydroxyl group (-OH) in the pullulan polysaccharide. The PUC polymer also showed a -C=O absorption peak at around 1700 cm^−1^. These characteristic peaks confirmed the successful synthesis of the PUC compound.

#### 2.1.2. NMR Hydrogen Spectroscopy for the Detection of PUC Polymers

The characteristic peaks for the α-1,4 glycosidic bond and the α-1,6 glycosidic bond of pullulan polysaccharide were observed at 4.60 ppm (1 Hα(1,6)) and 5.01 ppm (1 Hα(1,6)), respectively. The solvent peak (deuterated DMSO) at 2.51 ppm and the retention of the characteristic peaks for the pullulan polysaccharide in Figure 1B, along with the appearance of the absorption peak for -CH_2_CO- at 2.54 ppm, confirmed that CDCA was successfully grafted onto the pullulan polysaccharide chain and the PUC polymer was successfully synthesized. The degree of substitution (DS) of CDCA on pullulan polysaccharide was calculated using the following equation: DS = (Aδ_2.54_/2)/(Aδ_4.60_ + Aδ_5.01_).

The sum of the α-1,4 and α-1,6 glycosidic bonds in the pullulan polysaccharide corresponded to the number of pullulan units, as reported in the literature. Thus, the sum of the area of the two characteristic peaks (δ_4.60_, δ_5.01_) represented the total number of sugar units. Since there are two hydrogens in -CH_2_CO- in CDCA, the amount of CDCA can be calculated as one-half of Aδ_2.54_/2. Therefore, based on the above calculation, the degree of substitution of the PUC polymers with different feeding ratios was determined as shown in Table 1.

#### 2.1.3. Determination of the Particle Size and Zeta Potential of Nanoparticles

**Figure 2:**
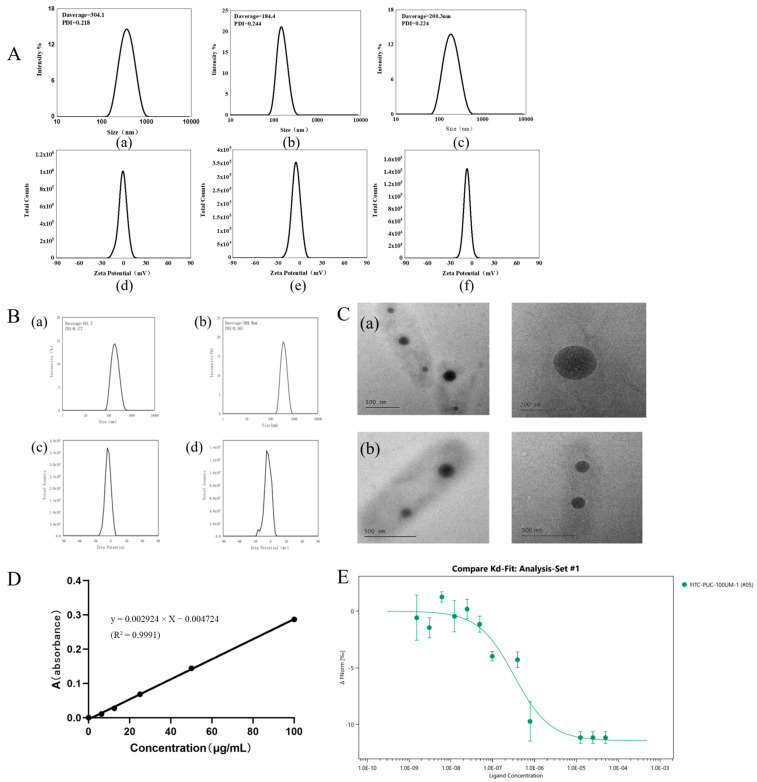
(**A**). Characterization of PUC nanoparticles at different feeding ratios by DLS; (**a**,**b**) shows the particle size and zeta potential of 0.13-scale nanoparticles; (**c**,**d**) shows the particle size and zeta potential of 0.15-scale nanoparticles; (**e**,**f**) shows the particle size and zeta potential of 0.18-scale nanoparticles. (**B**). (**a**,**b**) shows the particle size and potential values of adsorbed Tween-80-PUC nanoparticles; (**c**,**d**) are the particle size and potential values of BD-PUC. (**C**) Transmission electron microscopy observation of the morphology (**a**) for PUC nanoparticles; (**b**) for BD-PUC nanoparticles. (**D**). The standard curve for BD is y = 0.002924 × X − 0.004724 (R^2^ = 0.9991). (**E**). MST detection of Tween-80 and FITC-PUC nanoparticles by MST assay to verify the binding ability of both.

The particle sizes and zeta potentials of PUC nanoparticles, nanoparticles adsorbed with Tween-80, and nanoparticles encapsulated with BD at three different feeding ratios were 304.5 ± 3.669 nm, 184.1 ± 0.519 nm, and 196.6 ± 3.751 nm, respectively, for the 0.13, 0.15, and 0.18 feeding ratios (Figure 2A). At a feeding ratio of 0.13, the particle size of the nanoparticles was the largest. At a ratio of 0.15, the particle size was the smallest. And, at a feeding ratio of 0.18, the particle size of the nanoparticles increased again. Based on this result, it was speculated that the PUC polymer with a feed ratio of 0.15 reached the maximum hydrophilic upper limit. In contrast, the 0.13 PUC polymer, synthesized by reducing the feed ratio, was similar to the pullulan polysaccharide solution but with an increased particle size. When the feed ratio increased on this basis, the increase in the substitution of CDCA and the increase in steric hindrance led to an increase in the particle size of nanoparticles. The zeta potential of the PUC nanoparticles was negatively charged for all three degrees of substitution. All the above data has been summarized in Table 2.

The particle size change after adsorption of PUC nanoparticles by Tween-80 was minimal, with a size of 181.3 ± 2.984 nm. The nanoparticle size of PUC nanoparticles after BD loading was 381.1 ± 9.751 nm (Figure 2B). Nanoparticles loaded with biatractylolide may have had an increase in particle size due to the increase in hydrophobic core. According to scanning electron microscopy results (Figure 2C), PUC nanoparticles exhibited a spherical structure, and, after BD loading, the BD-PUC nanoparticles remained spherical. All the above data has been summarized in Table 3.

#### 2.1.4. Drug-Loading and Encapsulation Rates of Nanoparticles

The absorbance was measured using UV spectrophotometry, and 238 nm was selected as the detection wavelength of BD. The BD standard curve was plotted as shown in Figure 2D. The standard curve for BD was calculated as y = 0.002924 × X −0.004724 (R^2^ = 0.9991), and the concentration range was from 0 to 100 μg/mL. Based on this result, as shown in Table 4, the drug loading of the nanoparticles was calculated to be 3.17%, 3.74%, and 4.02% for three different dosing ratios (1:5, 1:10, and 1:20); their encapsulation rates were 50.81%, 59.48%, and 67.29%, respectively. The PUC polymer improved the water solubility of BD, increased the BD concentration in the nanosolution. and also offered more possibilities for BD formulation.

#### 2.1.5. MST Experiment

The microscale thermophoresis (MST) results (Figure 2E) showed that the thermal surge changed regularly with increasing Tween-80 concentration, yielding a KD value of 0.222 μM. This indicates that Tween-80 bound effectively to FITC-PUC, and the lower the KD value, the less material is required for binding, demonstrating a stronger binding affinity. These results indicate that Tween-80 has a strong binding capacity to FITC-PUC and the binding is stable. This demonstrates that Tween-80 can effectively bind to FITC-PUC nanoparticles, which will serve as the foundation for subsequent brain-targeting experiments.

### 2.2. In Vitro Experiments

#### 2.2.1. Cellular Uptake of PUC Nanoparticles

**Figure 3:**
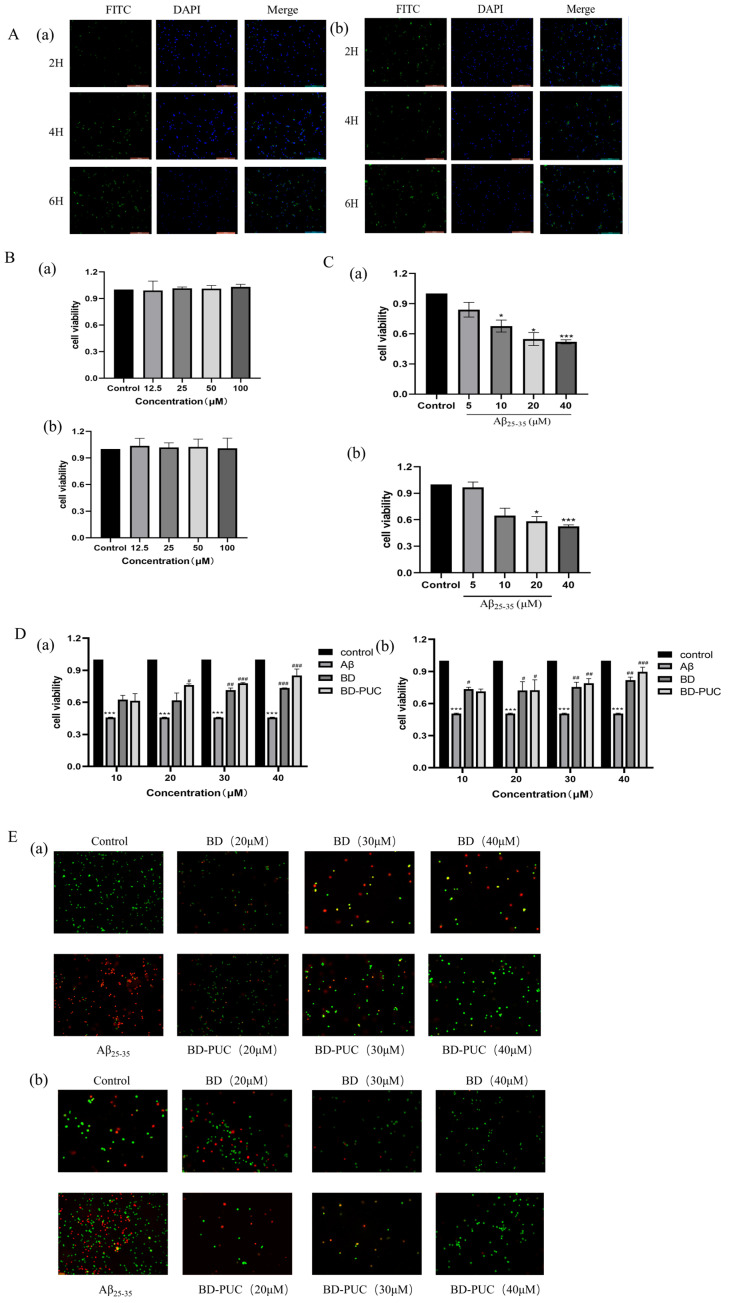
(**A**). After staining with DAPI and FITC-PUC nanoparticles: (**a**) inverted fluorescence images of SH-SY5Y cells, SH-SY5Y cells were able to take up PUC nanoparticles; (**b**) inverted fluorescence images of PC12 cells showed green fluorescence in PC12 cells, PC12 cells were able to take up PUC nanoparticles. (Scale length: 200 µm) (**B**). After treating cells with different concentrations of blank PUC nanoparticles: (**a**) PUC nanoparticles were not toxic to SH-SY5Y cells, (**b**) PUC nanoparticles were not toxic to PC12 cells. (**C**). After treatment of cells with different concentrations of Aβ_25-35_: (**a**) the effect of Aβ_25-35_ on the survival rate of SH-SY5Y cells, (**b**) the effect of Aβ_25-35_ on the survival rate of PC12 cells (*** *p* < 0.001 vs. control group; * *p* < 0.05 vs. control group). (**D**). Pretreatment of cells with different concentrations of BD-, BD-PUC-, Aβ_25-35_-simulated cell injury: (**a**) effect of BD-PUC on the activity of Aβ_25-35_-injured SH-SY5Y cells, (**b**) effect of BD-PUC on the activity of Aβ_25-35_-injured PC12 cell activity (*** *p* < 0.001 vs. control group; ^#^ *p* < 0.05 vs. Aβ_25-35_ group; ^##^ *p* < 0.01 vs. Aβ_25-35_ group; ^###^ *p* < 0.001 vs. Aβ_25-35_ group). (**E**). Pretreatment of cells with different concentrations of BD, BD-PUC, Aβ_25-35_ mock cell injury: (**a**) BD-PUC on apoptosis of Aβ_25-35_-injured SH-SY5Y cells, (**b**) BD-PUC on apoptosis of Aβ_25-35_-injured PC12 cells (green fluorescence: living cells; yellow fluorescence: early apoptosis; red fluorescence: late-stage apoptosis).

The uptake of FITC-PUC in SH-SY5Y and PC12 cells was observed using inverted fluorescence microscopy. The results (Figure 3A) showed that blue and green fluorescence overlapped in both cell lines, and the cells exhibited varying degrees of fluorescence uptake, indicating that FITC-PUC was internalized by SH-SY5Y and PC12 cells. Moreover, the green fluorescence in the cells was enhanced as the treatment time of FITC-PUC increased, indicating that the uptake of the nanoparticles by the cells increased with the treatment time. Based on this, it can be concluded that PUC nanoparticles can enter SH-SY5Y and PC12 cells, and the uptake rate increases with prolonged exposure.

#### 2.2.2. Effect of PUC Nanoparticles on the Viability of SH-SY5Y and PC12 Cells

The toxicity of blank PUC nanoparticles on SH-SY5Y cells and PC12 cells was detected by MTT assay (Figure 3B). As shown in the figure, there was no significant difference in cell viability between normal cells treated with various concentrations of blank PUC nanoparticles, indicating that these blank PUC nanoparticles were not significantly toxic to SH-SY5Y and PC12 cells.

#### 2.2.3. Establishment of Neural Cell Injury Model

The effect of Aβ_25-35_ on the viability of SH-SY5Y cells and PC12 cells was examined by the MTT method (Figure 3C). After cells were cultured in 96-well plates for 24 h and then exposed to different concentrations of Aβ_25-35_, the results showed a significant decrease in the cell viability of SH-SY5Y and PC12 cells as the concentration of Aβ_25-35_ increased. When the concentration of Aβ_25-35_ was 20 μM, the cell viability of SH-SY5Y cells and PC12 cells decreased to 0.478 ± 0.1367 and 0.506 ± 0.0036, which were significantly different from the control group. Therefore, a concentration of 20 μM Aβ_25-35_ was selected to establish the neural injury model in SH-SY5Y and PC12 cells.

#### 2.2.4. Effect of BD-PUC on the Viability of SH-SY5Y and PC12 Cells Injured by Aβ_25-35_ Induction

Different concentrations of free BD and BD-PUC were added to the well plates where the cells were cultured, and their effects on cell survival were assessed (Figure 3D). The results showed that the cell viability of SH-SY5Y cells treated with only 20 μΜ Aβ_25-35_ decreased to 0.4576 ± 0.0033 (*p* < 0.05) compared to the untreated group. In contrast, the viability of SH-SY5Y cells was significantly higher in the Aβ_25-35_-treated injury group compared to the spiked group, pretreated with different concentrations of BD and BD-PUC solutions, and cell viability was higher in the BD-PUC group than in the BD group (*p* < 0.05). The decrease in cell viability in the 20 μM Aβ_25-35_-treated group compared to the untreated group in PC12 cells was also significant, with the viability of PC12 cells in the Aβ_25-35_-treated injury group significantly higher than in the groups treated with different concentrations of BD and BD-PUC solutions, with the BD-PUC group showing higher cell viability than the BD group (*p* < 0.05).

#### 2.2.5. Effect of BD-PUC on Apoptosis of SH-SY5Y and PC12 Cells Injured by Aβ_25-35_ Induction

The changes in apoptosis of SH-SY5Y cells and PC12 cells were detected by using an AO and EB double-staining kit with different concentrations of BD and BD-PUC solutions (Figure 3E). Compared with the normal group, apoptotic cells increased significantly and red fluorescence intensity increased after SH-SY5Y cells were treated with 20 μΜ Aβ_25-35_. Compared with the Aβ_25-35_-treated group, green fluorescence intensity significantly increased and orange fluorescence intensity decreased in the groups pretreated with various concentrations of BD and BD-PUC, indicating a marked reduction in apoptotic cells following drug treatment. And the inhibition of cell damage was stronger in the BD-PUC-treated group than in the BD-treated group. In PC12 cells, the number of apoptotic cells significantly increased and cell damage was evident after treatment with 20 μΜ Aβ_25-35_. In contrast, compared with the Aβ_25-35_ damage group alone, pretreatment with BD and BD-PUC solutions significantly inhibited cell damage and improved cell morphology. And BD-PUC had a stronger ability to inhibit cell damage than the BD group did.

### 2.3. In Vivo Experiments

#### 2.3.1. In Vivo Imaging System to Observe the Brain-Targeting Effects of PUC

In order to verify that the nanoparticles adsorbed by Tween-80 have specific brain-targeting effects, mice were experimentally divided into three groups: the Tween-80-FITC-PUC group, the FITC-PUC group, and the control group without FITC.

**Figure 4:**
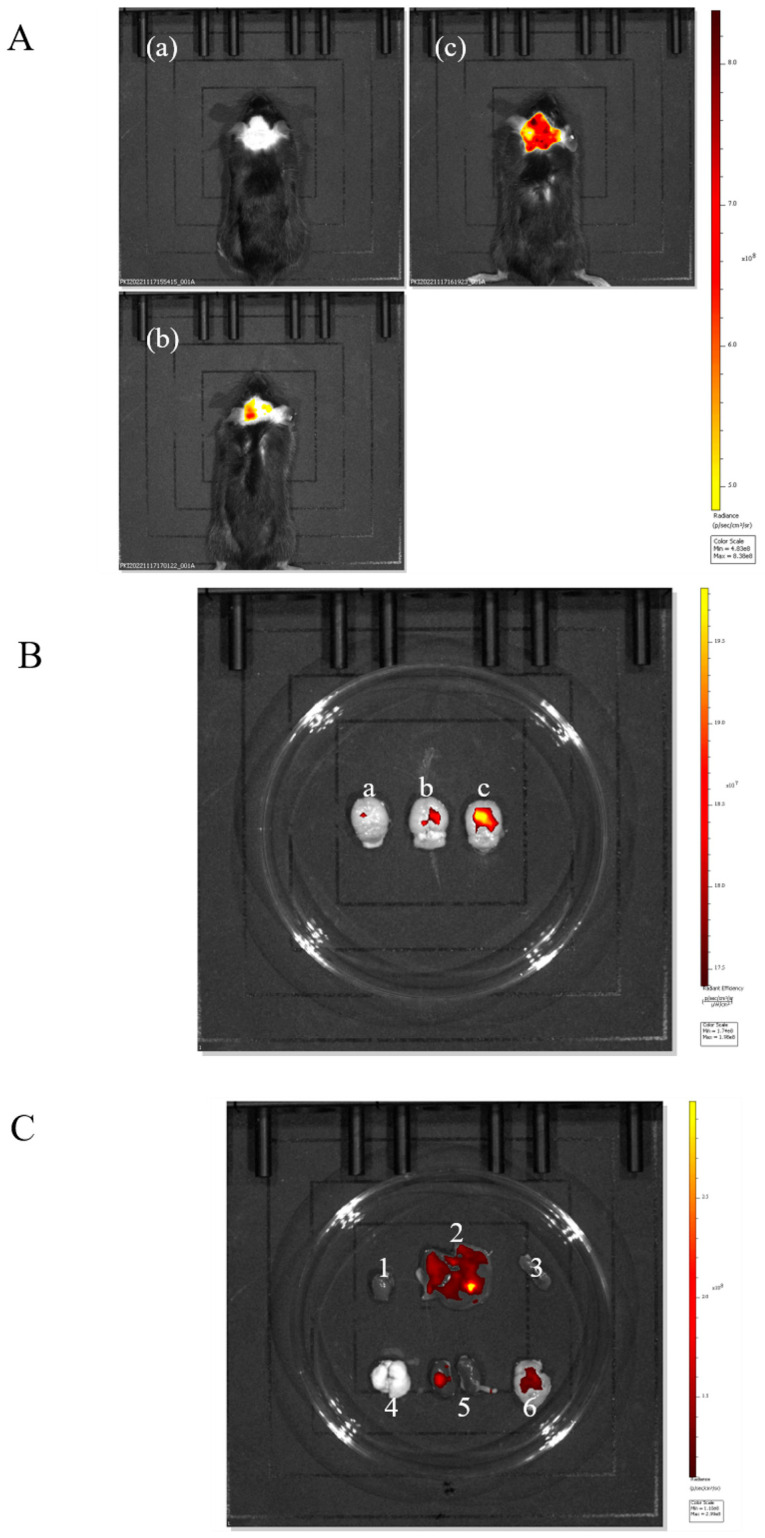
(**A**). In vivo imaging system for (**a**) free FITC injection, (**b**) FITC-PUC nanoparticle injection, (**c**) mice injected with Tween-80-modified FITC-PUC nanoparticles. (**B**). (**a**) Brain of mice injected with free FITC. (**b**) Brain of mice injected with FITC-PUC nanoparticles. (**c**) Brain of mice injected with FITC-PUC nanoparticles modified with Tween-80. (**C**). Imaging of organs of mice injected with Tween-80-modified FITC-PUC nanoparticles (1, 2, 3, 4, 5, 6 are heart, liver, spleen, lung, kidney, and brain, respectively).

As shown in Figure 4A, both the nanoparticles of the FITC-PUC group and those of the Tween-80-FITC-PUC group were able to reach the brain after injection into the tail vein compared with the free FITC group, indicating that these nanoparticles were able to cross the blood–brain barrier and enter the mouse brain.

We sampled the brains of the three groups of mice individually for comparison (Figure 4B). It was evident that the nanoparticles adsorbed with Tween-80 exhibited stronger fluorescence in the brain, clearly indicating that the Tween-80-modified nanoparticles had a brain-targeting effect.

To further verify the brian-targeting effect of the Tween-80-adsorbed nanoparticles, we observed the organ imaging of the Tween-80-FITC-PUC group (Figure 4C), which showed the presence of fluorescence in the liver, kidneys, and brain of the mice. Almost all drugs are eliminated through the liver and kidneys. We believe it is reasonable for fluorescence to accumulate in these organs, indicating that the nanoparticle was also metabolized and excreted through the liver and kidneys, without surprise.

#### 2.3.2. In Vivo Imaging System to Observe the Brain-Targeting Effect of BD-PUC

**Figure 5:**
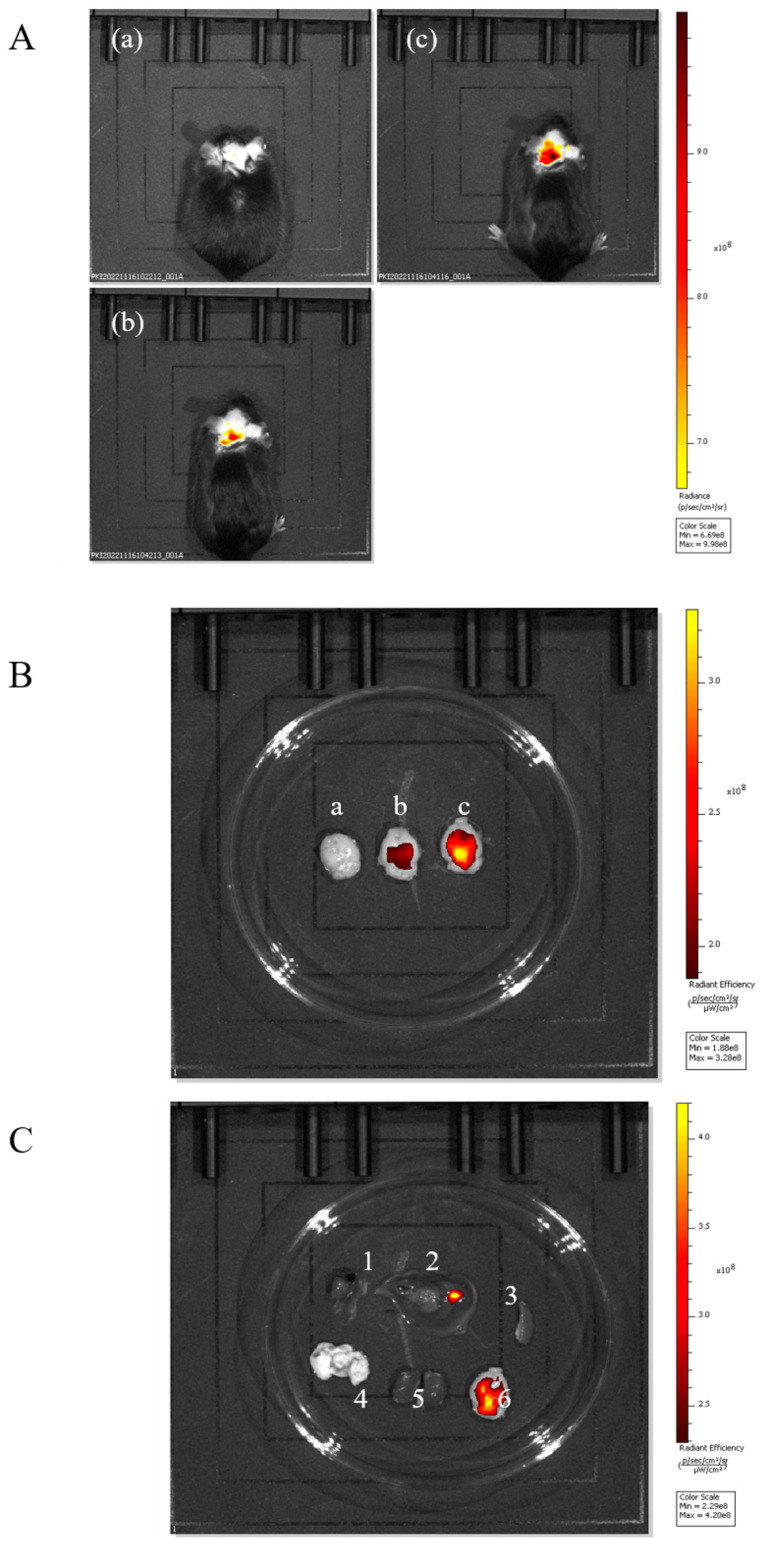
(**A**). In vivo imaging system assay: (**a**) mouse injected with free FITC; (**b**) mouse injected with FITC-BD-PUC nanoparticles; (**c**) mouse injected with Tween-80-modified FITC-BD-PUC nanoparticles. (**B**). In vivo imaging: (**a**) brain of mouse injected with free FITC; (**b**) brain of mouse injected with FITC-BD-PUC nanoparticles; (**c**) brain of mouse injected with Tween-80-modified FITC-BD-PUC nanoparticle mice. (**C**). Imaging of organs in mice injected with Tween-80-modified FITC-BD-PUC nanoparticles (1, 2, 3, 4, 5, 6 in the figure are heart, liver, spleen, lung, kidney, and brain, respectively).

The above experiments preliminarily confirmed the brain-targeting effect of Tween-80-PUC. Three groups of experiments in mice were designed to further validate the brain-targeting effect of the nanoparticles encapsulated with the biatractylolide on the brain. The three groups were the free FITC group, the FITC-BD-PUC group, and the Tween-80-FITC-BD-PUC group. As shown in Figure 5A,B, the in vivo fluorescence imaging results showed that the drug-encapsulated biatractylolide nanoparticles crossed the blood–brain barrier as well as the nanoparticles that were not encapsulated with the drug. In addition, the drug-loaded nanoparticles adsorbed with Tween-80 also maintained their targeting capability towards the brain. Furthermore, fluorescence is present in the liver, kidneys, and brain of the mice, which is consistent with the results from the previous section regarding the excretion of almost all drugs through the liver and kidneys (Figure 5C).

In addition, during the in vivo study, no signs of acute toxicity, behavioral changes, or hypersensitivity (e.g., skin erythema, piloerection, respiratory distress) were observed at the administered BD-PUC dose.

## 3. Discussion

The rising incidence of Alzheimer’s disease has taken a significant toll on the global population. Extensive research has been conducted on the development of new drugs and innovations in drug delivery for the treatment of Alzheimer’s disease. However, challenges such as intracranial drug delivery, low drug bioavailability, and limited targeting have emerged as major obstacles in Alzheimer’s disease drug development [28]. Therefore, humanity needs new strategies to treat Alzheimer’s disease.

In order to better treat Alzheimer’s disease, we propose a novel herbal extract compound, biatractylolide, as a therapeutic agent for Alzheimer’s disease. Biatractylolide is extracted from the traditional Chinese herb *Atractylodes macrocephala* and it was shown to possess therapeutic effects in Alzheimer’s disease [11]. For example, Zhao et al. [29] demonstrated that biatractylolide may counter Aβ-related neurotoxicity via anti-apoptotic and anti-inflammatory mechanisms, making it a promising candidate for AD therapy. Zhu et al. demonstrated at the cellular level that biatractylolide had a neuroprotective effect on glutamate-induced damage to PC12 and SH-SY5Y cells by inhibiting the PI3K-Akt-GSK3-dependent pathway [12].

Conventional therapies for treating AD have limited efficacy, partly because most drugs cannot cross the blood–brain barrier [30]. In recent studies, nanoparticles were proposed as an effective therapeutic approach for Alzheimer’s disease.

Lin et al. [31] developed hybrid cell membrane-coated liposomes combining platelet and chemokine (CCR2) cell membranes to enhance targeting. These liposomes were loaded with two different drugs (targeting distinct AD pathways) for synergistic therapy. The platelet/CCR2 membranes (bearing “self” CD47 signals) facilitated BBB crossing and homing to neuroinflammatory lesions. In conclusion, hybrid membrane-coated NPs provided precise, multi-targeted drug delivery in vivo. But this proof-of-concept work was preclinical, relying on a mouse model. The complex cell-membrane coating may pose manufacturing and immunological challenges for clinical translation.

Govindarajan and Kar [32] used native poly(lactic-co-glycolic acid) (PLGA) nanoparticles (a biodegradable polymer) as a theranostic agent. Fluorescently labeled PLGA NPs were injected into the brains of 5xFAD mice. The NPs co-localized with amyloid plaques: within 1–3 h after intracerebellar injection, labeled PLGA strongly bound both immunostained Aβ and Congo red-positive plaques in the cortex (peaking ~3 h post-injection). Importantly, no labeling occurred in wild-type controls. This was the first demonstration that drug-free PLGA NPs can selectively bind existing Aβ pathology in vivo, suggesting a role as “nano-theranostic” agents for AD. However, the study required direct brain injection (intracerebellar), which is not clinically practical. Systemic delivery strategies and the safety of repeated NP administration remain to be addressed.

Hou et al. [33] synthesized ultrasmall (3.3 nm) gold nanoparticles (AuNPs) capped with either L- or D-enantiomers of glutathione (L3.3 and D3.3) to probe stereospecific effects. Both AuNPs inhibited Aβ₄₂ fibril formation in vitro, but the D-glutathione AuNPs (D3.3) crossed the BBB more efficiently and accumulated longer in brain tissue. In APP/PS1 AD mice given weekly IV injections for 4 weeks, D3.3 treatment significantly improved spatial memory in the Morris water maze, whereas L3.3 showed no clear benefit. Immunostaining and ELISA revealed that both AuNPs lowered hippocampal Aβ levels. This study has its limitations: potential immunogenicity or off-target effects of metal NPs in humans remain to be explored.

Studies showed that nanoparticles are a new way to cross the blood–brain barrier by carrying drugs in the form of linkages or encapsulation, making the drugs more bioavailable and targeted with the help of nanocarriers [34]. Their unique physical and chemical properties enable targeted drug delivery to Alzheimer’s disease [35,36]. To facilitate drug delivery across the blood–brain barrier, we chemically synthesized Pullulan by linking it with small molecules of hydrophobic chenodeoxycholic acid to obtain Pullulan nanoparticles; using these nanoparticles to encapsulate the drug biatractylolide, PUC polymers with different feed ratios were prepared. Based on these preparations, we utilized the PUC nanoparticles in in-depth cellular studies to assess the reparative effects of nanoparticles on damaged cells. The results demonstrated that the biological activity of the drug biatractylolide, which was carried by nanoparticle PUC, was improved and had superior neuroprotective effect. To achieve brain targeting of the drug-loaded nanoparticles, Tween-80 was adsorbed on the surface of the nanoparticles. The experimental results showed that drug-loaded nanosolutions adsorbed with Tween-80 exhibited brain-targeting effects, confirming the feasibility of brain-targeted delivery using drug-loaded nanoparticles.

The nanoparticle formulation offers several compelling advantages: By significantly enhancing the solubility and stability of BD, it enables efficient, targeted delivery across the blood–brain barrier and consequently improves neuroprotective efficacy. Moreover, the versatile design of the carrier suggests it could be readily adapted to encapsulate other therapeutic agents. Furthermore, research conducted by Parveen et al. [37] demonstrated that, due to the inherent properties of nanoparticles, nanoparticle–protein interactions can occur, affecting the correct folding of unfolded or misfolded proteins and preventing their aggregation, which may contribute to the treatment of neurodegenerative diseases.

While many studies reported therapeutic benefits, nanoparticle safety is a critical concern. In general, small and negatively-charged NPs can penetrate the BBB, but their physicochemical properties also influence toxicity. For example, some metal-oxide NPs (e.g., TiO_2_, Fe_2_O_3_, NiO) were shown to exacerbate AD-like pathology by increasing oxidative stress, Aβ deposition, and neuronal apoptosis [38]. Nanoparticles may be recognized as ‘foreign’ by the immune system, triggering complement activation, cytokine release, or allergic reactions. Surface properties such as charge and hydrophobicity critically modulate this response [39]. Poorly biodegradable nanoparticles can accumulate in the liver, spleen, and other reticuloendothelial system organs, raising concerns about chronic toxicity [40]. Moreover, the crossing of nanoparticles through the blood–brain barrier is itself a risk that may lead to neurotoxicity.

This project represents a promising nanomedicine, providing an experimental foundation for neuroprotective drugs to exert efficient brain targeting to a certain extent. It also introduces a novel approach for Alzheimer’s disease drug therapy, with herbal medicine offering one of the most valuable resources for treating Alzheimer’s disease, thus bringing new hope to drug therapy for Alzheimer’s disease. The emergence of a nanodrug-targeted delivery system breaks through the limitations of traditional drug therapy and improves the blood–brain barrier permeability of Alzheimer’s disease drugs to a certain extent, which is expected to meet the increasing demand for Alzheimer’s disease diagnosis and treatment.

## 4. Materials and Methods

### 4.1. Materials

BD was extracted and purified by the School of Medicine, Hunan Normal University. The traditional Chinese medicinal material *A. macrocephala* (Baizhu) was extracted using ethyl acetate, and modern chromatography was employed to separate the *A. macrocephala* extract to obtain BD.

The air-dried root of *A. macrocephala* (15 kg, comes from Pan’an County, China) was extracted three times at room temperature over seven days with ethyl acetate (EtOAc). The combined EtOAc extracts were concentrated under reduced pressure to give a dark-green residue (275 g). This residue was dissolved in a mixture of petroleum ether and EtOAc and then partitioned between the two solvents. The petroleum ether fraction was subjected to column chromatography on silica gel and eluted stepwise with petroleum ether–EtOAc *volume:volume* 20:1, 10:1, 10:2, 10:3). The fraction eluted with petroleum ether–EtOAc (10:1) was further purified by repeated silica gel column chromatography (petroleum ether–acetone, *volume:volume* 20:1 → 5:2) and preparative thin-layer chromatography (TLC), affording biatractylolide (480 mg). This method is capable of reproducibly extracting the pure compound biatractylolide.

The reagents used in this experiment are listed in Table 5 and the instruments used in this experiment are listed in Table 6.

### 4.2. Cell Culture

PC12 cells (rat adrenal pheochromocytoma cells) were obtained from Wuhan Ponosei Life Science and Technology Co., Ltd. (Wuhan, China) and SH-SY5Y cells (human neuroblastoma cell line) were sourced from the Medical College of Hunan Normal University. Cells were cultured in DMEM medium supplemented with 10% fetal bovine serum and 1% antibiotics (penicillin-streptomycin) and maintained in a humidified incubator at 37 °C with 5% CO_2_.

### 4.3. Animal

C57 BL/6J (6–8 weeks, 18–22 g, male/female = 1) mice were selected as the experimental model, and the mice were purchased from Jiangsu Jichui Yaokang Biotechnology Co., Ltd. (Nanjing, China) Experimental protocols were approved by the Biomedical Research Ethics Committee of Hunan Normal University (D2022052, approval date: 26 August 2022). All animals were handled in accordance with institutional animal care and use committee (IACUC) regulations.

### 4.4. Characterization of Nanoparticles

#### 4.4.1. Preparation of PUC Polymers

**Figure 6:**
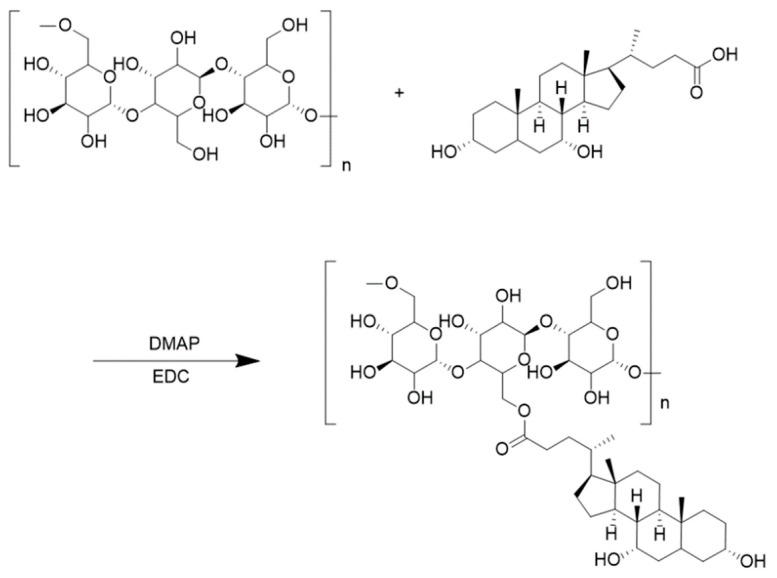
PUC polymer synthesis chemical reaction formula.

Three g of pullulan polysaccharide powder was dissolved in thirty mL DMSO for later use. Then, according to the feeding ratio, three different amounts of deoxycholic acid (PUL: CDCA = 0.13, 0.15, or 0.18 mmol/mmol), 4-dimethylaminopyridine (DMAP), and 1-(3-dimethylaminopropyl)-3-ethyl hydrochloric acid carbodiimide (CDCA/EDC/DMAP = 1:2:1 mmol/mmol) were measured, dissolved in 15 mL dimethyl sulfoxide, and pre-stirred at 37 °C for 2 h. The pullulan polysaccharide solution was slowly dropped into the activation solution, transferred to a round-bottomed flask, and continued to react in an oil bath at 50 °C for 48 h. After the reaction, the reacted solution was dropped into anhydrous ethanol to obtain a white precipitate, which was filtered using extraction filter. Three dried PUC polymers with different substitution degrees (0.13, 0.15, 0.18) were obtained after volatilization of anhydrous ethanol. Figure 6 presents the chemical reaction formula for the synthesis of PUC polymers. Ten mg of PUC polymer was dissolved in DMSO-d6 and analyzed by ^1^H-NMR spectra. The extent of CDCA substitution on Pullulan was determined by the area under -1,4 glycosidic bonds, -1,6 glycosidic bonds and n-butyl signals.

#### 4.4.2. Preparation of PUC Nanoparticles

PUC nanoparticles were prepared using a water-dialysis method. Modified pullulan (20 mg, at different feed ratios) was dissolved in 2 mL DMSO. The solution was placed in a dialysis bag (Molecular weight cut-off: 3000 Da) and dialyzed against 500 mL deionized water. During the first 24 h, the water was changed every 3 h and then every 6 h during the next 24 h. After 48 h, DMSO was fully removed. The resulting dispersion was sonicated at 50 W for 2 min to obtain blank PUC nanoparticles, which were stored at 4 °C.

#### 4.4.3. Preparation of BD-Loaded PUC Nanoparticles

BD-loaded PUC nanoparticles were prepared similarly. PUC (20 mg) and BD were dissolved in DMSO at feed ratios of 1:5, 1:10, and 1:15 (BD:PUC, *w*/*w*) until fully dissolved. The solution underwent the same dialysis procedure as above: 48 h against 500 mL deionized water with water changes every 3 h (first 24 h) and every 6 h (next 24 h). After dialysis, the dispersion was sonicated at 50 W for 2 min and filtered through a 0.45 μm membrane. The resulting BD-PUC nanoparticles were stored at 4 °C.

#### 4.4.4. Nanoparticle Morphology and Nanoparticle Size and Zeta Potential

The size, polymer dispersity index (PDI), and zeta potential of PUC, BD-PUC, and Tween-80-BD-PUC nanoparticles with different proportions were analyzed by dynamic light scattering method (DLS). The average particle size and particle size distribution of the obtained uniform suspension were measured three times.

The shape, surface morphology, and size of PUC and BD-PUC nanoparticles were observed by transmission electron microscopy. The PUC and BD-PUC were placed on a carbon-coated copper net to form a thin, liquid film. After the film was naturally dried, the samples were negatively stained with 2% (*w*/*v*) tungsten phosphide acid solution, and the surface structure was observed under transmission electron microscope.

#### 4.4.5. Nanoparticle Loading and Encapsulation Rates

The standard curve of BD was established by UV-vis spectrophotometer. The drug-loading capacity (LC %) and encapsulation efficiency (EE %) of BD-loaded PUC nanoparticles loaded were determined using the following method: 4 mL of freshly prepared BD-PUC nanoparticles with three different feeding ratios were analyzed for absorbance using the UV-vis spectrophotometer. Blank PUC nanoparticles with the same solvent were used as blank group. The drug content was calculated according to the standard curve, and then the drug-loading and encapsulation rates were obtained according to the following formula:

(*W_loaded_*: the weight of the drug in the nanoparticles; *W_NPs_*: the weight of the drug-loaded nanoparticles; *W_added_*: the weight of the drug used in the preparation of the nanoparticles)

$$Loading\;capacity\;(LC\%)=W_{loaded}/W_{NPs}\times 100\%$$

$$Encapsulation\;efficiency\;\left(EE\%\right)=W_{loaded}/W_{added}\times 100\%$$

### 4.5. Cell Experiments

#### 4.5.1. Cellular Uptake

PC12 and SH-SY5Y cells were inoculated into 12-well plates with cell density of 5 × 10^4^ cells per well. The cells were incubated at constant temperature for 24 h. Then, 200 μL FITC-BD-PUC nanoparticles were added to 12-well plates, and the cells were treated for 2 h, 4 h, and 6 h, respectively. After the liquid was absorbed, 500 μL of 4% paraformaldehyde was added to each well to fix the cells. After treatment for 15 min, the fixing solution was removed. Next, 10 μL DAPI was added for nuclear staining, and the excess DAPI solution was washed off after 10 min of incubation. A coverslip was applied to the rectangular slide, which was then inverted onto the slide. The slide was sealed with nail polish. Photographs were taken using an inverted fluorescence microscope with blue light excitation.

#### 4.5.2. MTT Assay for Cell Viability

SH-SY5Y and PC12 cells at logarithmic growth stage were inoculated into 96-well plates with a density of about 5 × 10^3^ cells per well. Sterile PBS was added around the edges of the plates. After 24 h, when the cells had adhered, different concentrations of drugs (the final DMSO concentration did not exceed 0.1% (volume:volume) were added, and the culture was continued for 48 h. The cells were observed under an inverted microscope. Then, 20 μL MTT (5 mg/mL) was added to each well and cultured in a constant temperature incubator for 4 h. Formazan was fully dissolved in DMSO, and the absorbance was measured at 490 nm with the blank control set to zero. The absorbance (A) of each hole at 490 nm wavelength was detected by enzyme-labeled instrument. Relative cell viability = [(experimental hole − blank hole)/(control hole − blank hole)] × 100%. Each group was tested in triplicate.

#### 4.5.3. AO/EB Staining Method to Observe Apoptosis Morphology

SH-SY5Y and PC12 cells at logarithmic growth stage were taken and inoculated in 6-well plates at a density of approximately 5 × 10^5^ and 8 × 10^5^ cells per well, respectively. After 24 h of incubation, SH-SY5Y cells and PC12 cells were incubated for 24 h. The supernatant was discarded after 24 h, and the cells were stained using the AO/EB staining kit. The cells were protected from light and observed for morphological changes.

#### 4.5.4. Statistical Processing and Data Analysis

All experiments were repeated three times, and the results were expressed as mean ± standard deviation. Data were statistically analyzed using GraphPad Prism (V9.3) software, one-way ANOVA, Student’s *t*-test, and the least squares method. The results were considered statistically significant at *p* < 0.05.

### 4.6. Animal Experiments

#### 4.6.1. Tween-80 Dilution Series and Nanoparticle Solubility Measurement via MST

Tween-80 was dissolved in dH_2_O and diluted to 100 μM. Next, 10 μL reagent 1 was added to centrifuge tube 2, followed by 10 μL dH_2_O to dilute reagent 2. Then, 10 μL of reagent 2 was added to centrifuge tube 3, and 10 μL dH_2_O was added to dilute reagent 3. A total of 16 tubes of graded dilutions of Tween-80 were prepared as described. Then, 10 μL of each concentration was added to EP tubes labeled 1 through 16. Next, 10 μL of the appropriate solubility of FITC-PUC nanoparticle solution was added to each tube and mixed. The MST data were analyzed using NT-analysis software, and the KD value was fitted according to the mass action law as described in the software.

#### 4.6.2. In Vivo Fluorescence Imaging to Observe the Brain-Targeting Effects of Nanoparticles

To verify that the fluorescent nanoparticles FITC-PUC adsorbed with Tween-80 can target the brain, healthy C57 BL/6J mice (6–8 weeks, 18–22 g, male/female = 1) were randomly divided into three groups: the Tween-80-FITC-PUC injection group, the FITC-PUC group, and the control group injected with free FITC. Each group of mice was injected with 200 μL of the corresponding drug via the tail vein and anesthetized with an intraperitoneal injection of 0.4% sodium pentobarbital (50 mg/kg) at 0.5 h after the injection. Once the mice were fully anesthetized, they were placed on the imaging platform of the in vivo imager, and fluorescence imaging was performed using the appropriate wavelengths to obtain a fluorescence imaging map to observe the fluorescence targeting the brain of the mice. After in vivo imaging, the mice were euthanized (Pentobarbital Sodium, i.v.), and their organs (heart, liver, spleen, lungs, kidneys, and brain) were collected for further fluorescence imaging to observe the distribution of fluorescence. This experiment was repeated three times.

#### 4.6.3. In Vivo Fluorescence Imaging to Observe the Brain-Targeting Effects of Drug-Loaded Nanoparticles

To verify the brain-targeting ability of Tween-80 adsorbed onto nanoparticles loaded with the drug bilobalide, a similar experiment was designed. Healthy C57 BL/6J mice (6–8 weeks, 18–22 g, male/female = 1) were randomly divided into three groups. The groups were divided as follows: Tween-80-injected-FITC-BD-PUC group, FITC-BD-PUC group, and control group injected with free FITC. The experimental methods used for in vivo fluorescence imaging were identical to those described above.

## 5. Conclusions

As we mentioned in the discussion section, in this study, we successfully synthesized Pullulan-modified polymers. The protective effect of nanoparticles encapsulating the drug biatractylolide on Aβ_25-35_-induced damage in SH-SY5Y and PC12 cells was confirmed to be superior to that of a free biatractylolide solution. And the brain-targeting effect of BD-PUC nanoparticles adsorbed with Tween-80 was verified to improve the bioavailability of the drug in vivo, which provides a new theoretical basis for the treatment of Alzheimer’s disease. However, despite these promising results, meeting the growing demand for the effective diagnosis and treatment of Alzheimer’s disease remains a significant challenge.

This study still requires further in-depth investigation. In further research, the long-term toxicity studies of nanoparticles and the mechanisms of brain targeting need to be considered.

## Figures and Tables

**Table 1:** Degree of substitution of PUC polymers with different feeding ratios.

Dosing Ratio	0.13	0.15	0.18
Hydrophobic substitution	8.97%	10.66%	13.92%

**Table 2:** Particle size, potential, and PDI of PUC with different feeding ratios.

PUC	Size (nm)	PDI	Zeta (mV)
0.13 PUC	304.5 ± 3.669	0.206 ± 0.016	−1.14 ± 0.225
0.15 PUC	184.1 ± 0.519	0.194 ± 0.043	−5.12 ± 0.290
0.18 PUC	196.6 ± 3.751	0.228 ± 0.024	−6.18 ± 0.490

**Table 3:** Particle size, potential, and PDI of Tween-80-PUC and BD-PUC.

	Size (nm)	PDI	Zeta (mV)
Tween80-PUC	181.3 ± 2.984	0.177 ± 0.004	−4.91 ± 0.264
PUC-BD	381.1 ± 9.751	0.113 ± 0.046	−5.12 ± 0.290

**Table 4:** LC % and EE % of drug-loaded nanoparticles with three different dosing ratios.

PUC/BD	5:1	10:1	20:1
LC %	3.17%	3.74%	4.02%
EE %	50.81%	59.48%	67.29%

**Table 5:** Reagents used in this study.

Reagent	Manufacturer	Purity
BD	Homemade by Hunan Normal University, Changsha, China	N/A
Aβ_25-35_	Sigma Chemicals, St. Louis, MO, USA	≥97% (HPLC)
DMSO	Sigma Chemicals, St. Louis, MO, USA	≥99.5%
Chenodeoxycholic acid	Shanghai Macklin Biochemical Co., Ltd., Shanghai, China	≥98%
Pullulan	Aladdin Scientific, Shanghai, China	N/A
Tween-80	Tianjin Fuchen Reagent Research Institute, Tianjin, China	N/A
FITC	Shanghai Yuanye Biotechnology Co., Ltd., Shanghai, China	≥97% (HPLC)
MTT	Sigma Corporation, Shanghai, China	≥97%
Newborn bovine serum	Gibco, Shanghai, China	N/A
DAPI	Biyuntian Company, Shanghai, China	N/A
AO/EB Double dye fluorescence kit	Sinopsin Chemical Reagent Co., Ltd., Shanghai, China	N/A

**Table 6:** Experimental instruments used in this study.

Experimental instruments	Manufacturer	Model
Ultraviolet spectrophotometer	SHIMADZU, Kyoto, Japan	UV-Probe-2450
Fourier transform infrared spectrophotometer	Thermo-Fisher, Madison, WI, USA	Nicolet-360
Transmission electron microscope	FEI, Hillsboro, OR, USA	N/A
Laser particle size analyzer	Malvern Panalytical, Malvern, UK	Zetasizer Nano
MRI machine	Bruker, Billerica, MA, USA	500 MHz
Inverted fluorescence microscope	Leica, Weztlar, Germany	DMI3000B
Multimode microplate reader	BioTeK, Winooski, VT, USA	Synergy HTX
Small-animal in vivo imaging system	PerkinElmer, Shelton, CT, USA	IVIS Lumina LT

## Data Availability

The original contributions presented in this study are included in the article. Further inquiries can be directed to the corresponding authors.

## Abbreviations

The following abbreviations are used in this manuscript:

CDCA	Chenodeoxycholic acid
PUC	Pullulan
Tween-80	Polysorbate 80
AD	Alzheimer’s disease
ChAT	Choline acetyltransferase
Ach	Acetylcholine
Aβ	Amyloid-beta
BD	Biatractylolide
BBB	Blood–brain barrier
LDL	Low-density lipoprotein
FITC	Fluorescein isothiocyanate
MST	Microscale thermophoresis
MTT	Methylthiazolyldiphenyl-tetrazolium bromide
DMSO	Dimethyl sulfoxide
